# Cardiovascular and neurovascular complications in systemic lupus Erythematosus: A cross-sectional study of subclinical atherosclerosis and cognitive dysfunction

**DOI:** 10.6026/973206300214241

**Published:** 2025-11-15

**Authors:** Abberamiy Pushparaj, Saradha Muthu Kumar Padmasini, Akhila Pakalapati, Sorabh Sharma

**Affiliations:** 1 Department of Gastro and Rheumatology, Ramchandra Hospital, Sri Lanka, South Asia; 2 Department of Critical Care, Christian Medical College, Vellore, Tamil Nadu, India; 3 Department of Internal Medicine, King's College London, Minnesota, USA; 4 Department of Internal Medicine, University of Arizona, Arizona, USA

**Keywords:** Systemic lupus erythematosus, subclinical atherosclerosis, cognitive dysfunction, carotid intima-media thickness, MoCA, neurovascular complications

## Abstract

Systemic Lupus Erythematosus (SLE) is a chronic autoimmune condition that has increase appreciated cardiovascular and neurovascular complications. This report presents a cross-sectional study of 100 SLE patients (18-55 years) who were evaluated for the prevalence and relationship of subclinical atherosclerosis and cognitive dysfunction. Subclinical atherosclerosis was measured by carotid intima-media thickness (CIMT) and cognition was measured by scoring the Montreal Cognitive Assessment (MoCA). The study found that 36% of participants had subclinical atherosclerosis, while 42% had cognitive impairment. Both subclinical atherosclerosis and cognitive dysfunction were associated with longer disease duration, corticosteroid exposure, higher SLEDAI scores and the presence of antiphospholipid antibodies. Data shows the high prevalence of concomitant vascular and cognitive dysfunction in SLE patients and the need for early screening for both outcomes in a non-invasive manner.

## Background:

Systemic Lupus Erythematosus (SLE) is a long-lasting autoimmune condition that features systemic inflammation, deposition of immune complexes and multi-organ involvement. Immunosuppressive treatments have improved short-term mortality; however long-term complications, particularly cardiovascular and neuropsychiatric complications, continue to be a central contributor to morbidity and mortality [1]. Cardiovascular disease develops on average 15 years before the general population and the risk of myocardial infarction is estimated to be up to 50-fold higher among women with lupus at a younger age [1]. Atherosclerosis is considered "accelerated" atherosclerosis in this group. This is thought to result from an intersection of on-going immune activation, endothelial dysfunction and oxidative stress and pro-thrombotic antiphospholipid antibodies [3]. More traditional cardiovascular risk factors alone do not explain the increased burden of vascular disease; therefore, mechanisms specific to the disease can be postulated. Chronic inflammation leads to endothelial cell activation and vascular remodeling and autoantibodies contribute to platelet aggregation, as well as facilitate anticoagulant pathways [4]. Several mechanisms specific to lupus have been discovered and imaging by carotid ultrasound and assessment of carotid intima-media thickness (CIMT) can be effective for determining subclinical atherosclerosis in asymptomatic patients [5]. A number of studies indicate that higher CIMT and plaque burden is correlated to lupus disease activity and duration, suggesting that patients with SLE undergo an additive process specific to vascular injury across time [6]. Neuropsychiatric lupus (NPSLE) is another serious clinical problem in addition to cardiovascular morbidity. Neuropsychiatric manifestations are observed in approximately 30-40% of patients, ranging from headaches and mood disorders to seizures, psychosis and cognitive dysfunction [7]. Cognitive impairment has, in particular, recently gained recognition for being a frequent and disabling symptom, as patients may experience memory loss, diminished concentration, or executive dysfunction, all of which may occur independently of obvious neuropsychiatric events. Mechanistic studies suggest cognitive impairment in SLE is multifactorial with references to small-vessel ischemia, antibody-mediated neuronal injury and chronic neuroinflammation [8]. The recognition of overlap between vascular pathology and cognitive decline in SLE has occurred more recently. For example, subclinical atherosclerosis may precipitate cerebral hypoperfusion and microvascular injury, worsening neurocognitive symptoms [9]. Antiphospholipid antibodies, in addition to playing a role in thrombosis, have been linked to direct neuronal injury and synaptic dysfunction adding links between vascular and neuropsychiatric mechanisms. As such, early diagnosis of subclinical vascular disease could provide important information on cognitive prognosis, while improving the management of disease management and reducing the risk of neuropsychiatric manifestations [10]. Serological markers and treatment regimens also appear to impact these endpoints. Elevated inflammatory cytokines, circulating immune complexes and markers of endothelial activation have all been linked with both CIMT progression and cognitive decline [11]. However, while chronic exposure to corticosteroids is effective for managing disease flares, it is also associated with dyslipidemia, hypertension and insulin resistance, all of which exacerbate cardiovascular risk. On the other hand, hydroxychloroquine has been linked with vascular protection and improved lipid profiles which would indicate that it has effects beyond immunomodulation [12].

Despite these advancements, there are still significant gaps in our understanding of how subclinical vascular changes result in cognitive dysfunction in SLE. Most studies have examined cardiovascular or neuropsychiatric outcomes in isolation and have not fully analyzed the overlap of these two medical phenomena [13]. In addition, cognitive dysfunction due to lupus is often overlooked whereby symptoms blend with fatigue and depression and since clinicians do not routinely conduct standardized neuropsychological assessments, these cognitive issues are frequently unnoticed [14]. Therefore, the prevalence is cited widely without real certainty of the impact of cognitive dysfunction on SLE. Understanding the relationship between vascular pathology and neurocognitive outcomes is imperative for future evaluation of stratifying risk and implementing personalized management plans [15]. Expanding studies to monitor serological markers, vascular imaging and baseline cognitive testing in longitudinal studies may help support the causal pathways to evaluate modifiable risk factors [16]. In conclusion, an integrated approach may facilitate earlier intervention to optimize cardiovascular and neurological outcomes in this at-risk population [17]. Therefore, it is of interest to characterize the prevalence and association between subclinical atherosclerosis and cognitive impairment in patients with SLE while also evaluating the clinical, serological and treatment factors which may contribute to these outcomes.

## Materials and Methods:

## Study design and population:

This was a cross-sectional observational study conducted at a tertiary care hospital over 18 months. A total of 100 patients diagnosed with SLE, fulfilling the 2019 ACR/EULAR classification criteria, were enrolled consecutively.

## Inclusion criteria:

[1] Age 18-55 years

[2] Diagnosed with SLE for ≥6 months

[3] Stable clinical condition (no acute flare)

## Exclusion criteria:

[1] History of cerebrovascular accident, myocardial infarction, or psychiatric illness

[2] Diabetes mellitus or uncontrolled hypertension

[3] Chronic kidney disease (eGFR <60 ml/min/1.73 m^2^)

[4] Active infection or malignancy

## Data collection:

A structured proforma was used to collect:

[1] Demographics: Age, sex, BMI, smoking, comorbidities

[2] Clinical: Disease duration, SLEDAI-2K score, corticosteroid and immunosuppressive use

[3] Laboratory: ANA, anti-dsDNA, complement (C3/C4), lipid profile, antiphospholipid antibodies

[4] Subclinical Atherosclerosis: Measured by carotid Doppler ultrasound; CIMT >0.8 mm was considered abnormal

[5] Cognitive Function: Assessed using MoCA; score <26 indicated impairment

## Statistical analysis:

Data were analyzed using SPSS v25. Continuous variables were expressed as mean ± SD. Categorical variables were presented as percentages. Correlation between variables was analyzed using Pearson or Spearman correlation tests. A p-value <0.05 was considered significant.

## Results:

The study included 100 patients diagnosed with Systemic Lupus Erythematosus (SLE), with a mean age of 32.4 ± 7.8 years. The majority of the study populations were female (88%) and the mean duration of disease since diagnosis was 5.8 ± 3.4 years. The average SLE Disease Activity Index (SLEDAI-2K) score was 8.2 ± 3.1, indicating mild to moderate disease activity. Hypertension and dyslipidemia were present in 27% and 34% of patients, respectively. Antiphospholipid antibody (APS) positivity was observed in 22% of the cohort. Subclinical atherosclerosis, defined by a carotid intima-media thickness (CIMT) greater than 0.8 mm, was identified in 36% of patients. Those with subclinical atherosclerosis had a significantly longer disease duration (p = 0.004), higher low-density lipoprotein (LDL) cholesterol levels (p = 0.01) and were more frequently APS-positive (p = 0.03). Cognitive dysfunction, indicated by Montreal Cognitive Assessment (MoCA) scores below 26, was found in 42% of patients. The most commonly affected domains were attention and concentration (61%), followed by memory recall (52%) and executive function (41%). Cognitive impairment was significantly associated with higher cumulative corticosteroid use (p = 0.02), low complement C3 levels (p = 0.01) and anti-dsDNA positivity (p = 0.04). Importantly, 24 patients (24%) exhibited both subclinical atherosclerosis and cognitive dysfunction. These patients demonstrated significantly higher SLEDAI-2K scores, with a mean score of 10.1 ± 2.7 and had a higher prevalence of APS positivity compared to others (p < 0.01), indicating a possible shared pathogenic mechanism or cumulative disease burden. Table 1 (see PDF) shows the baseline demographic and clinical characteristics of SLE patients, including mean age, sex distribution, disease duration, disease activity score and comorbid conditions. Table 2 (see PDF) presents the prevalence of subclinical atherosclerosis and its associated factors, highlighting significant associations with longer disease duration, higher LDL cholesterol levels and APS positivity. Table 3 (see PDF) shows cognitive dysfunction and related clinical factors, with significant associations observed for higher cumulative steroid dose, low complement C3 levels and anti-dsDNA positivity. Table 4 (see PDF) compares patients with both subclinical atherosclerosis and cognitive dysfunction against others, indicating significantly higher SLEDAI scores and APS positivity in the dual-complication group. The study included 100 patients with Systemic Lupus Erythematosus (SLE), with a mean age of 32.4 ± 7.8 years. The majority were female (88%) and the average disease duration was 5.8 ± 3.4 years. The mean SLE Disease Activity Index (SLEDAI-2K) score was 8.2 ± 3.1, reflecting mild to moderate activity. Hypertension and dyslipidemia were observed in 27% and 34% of patients, respectively, while antiphospholipid antibody (APS) positivity was present in 22% (Table 1 - see PDF). Subclinical atherosclerosis, defined as carotid intima-media thickness (CIMT) >0.8 mm, was identified in 36% of patients. Those with atherosclerosis had a significantly longer disease duration (p = 0.004), higher LDL cholesterol levels (p = 0.01) and more frequent APS positivity (p = 0.03) compared to patients without atherosclerosis (Table 2 - see PDF). Cognitive dysfunction, defined as a Montreal Cognitive Assessment (MoCA) score <26, was present in 42% of the cohort. The most affected cognitive domains included attention and concentration (61%), memory recall (52%) and executive function (41%). Cognitive impairment was significantly associated with higher cumulative corticosteroid exposure (p = 0.02), low complement C3 levels (p = 0.01) and anti-dsDNA positivity (p = 0.04) (Table 3 - see PDF). Importantly, 24 patients (24%) exhibited both subclinical atherosclerosis and cognitive dysfunction. Compared with the remaining patients, this subgroup demonstrated significantly higher mean SLEDAI-2K scores (10.1 ± 2.7) and greater APS positivity (p < 0.01), suggesting a possible shared pathogenic mechanism or additive disease burden (Table 4 - see PDF).

## Discussion:

The present study highlights the substantial burden of subclinical cardiovascular and neurocognitive complications among individuals with Systemic Lupus Erythematosus [4]. The prevalence of subclinical atherosclerosis observed in this cohort, reflected by increased carotid intima-media thickness, underscores the presence of accelerated vascular aging even among relatively young patients [5]. Similarly, the high frequency of cognitive impairment identified using the Montreal Cognitive Assessment reinforces the growing clinical recognition that neurocognitive deficits in lupus are common, often subtle and frequently underdiagnosed during routine clinical visits. The observation that both subclinical atherosclerosis and cognitive dysfunction were significantly associated with longer disease duration, higher disease activity scores and the presence of antiphospholipid antibodies supports the concept that cumulative inflammatory and thrombotic burden contributes to progressive vascular and neurological injury. The finding that nearly one-fourth of the patients demonstrated both complications simultaneously further strengthens the likelihood of shared pathogenic pathways involving chronic endothelial damage, microvascular dysfunction and sustained immune-mediated injury [18]. Corticosteroid exposure emerged as an important factor linked to cognitive deficits and vascular abnormalities. This aligns with known metabolic and vascular effects of long-term steroid therapy, including dyslipidemia, hypertension and increased oxidative stress. These findings reinforce the clinical importance of adopting steroid-sparing regimens whenever feasible. Conversely, the protective influence of better-controlled disease activity and favourable serological profiles supports the value of early and sustained disease control to mitigate long-term complications [19]. The pattern of cognitive deficits in this study particularly affecting attention, memory and executive functioning highlights the need for systematic neurocognitive screening in SLE. Such deficits may significantly affect daily functioning despite the absence of overt neuropsychiatric events. Similarly, the identification of subclinical atherosclerosis emphasizes the utility of non-invasive vascular imaging as part of comprehensive patient assessment, especially given that traditional cardiovascular risk factors alone do not fully explain the elevated vascular risk in lupus [20]. The combined presence of abnormal vascular markers and cognitive impairment suggests that early vascular changes may contribute to cerebral hypoperfusion, subtle ischemia and eventual neurocognitive decline. This interrelationship supports the need for integrated evaluation strategies that simultaneously address cardiovascular and neurovascular health rather than managing these domains in isolation. Moreover, the associations with antiphospholipid antibodies point toward a possible thrombogenic contribution to both carotid pathology and cognitive dysfunction, warranting closer monitoring of individuals with positive serological markers [21]. Overall, the findings emphasize the importance of proactive screening, early intervention and individualized risk modification strategies. Incorporating CIMT measurement and MoCA testing into routine care may provide valuable opportunities for earlier detection of high-risk patients, enabling timely interventions to limit progression of vascular and cognitive complications.

## Conclusion:

Subclinical cardiovascular and cognitive abnormalities are frequent and underdiagnosed in SLE patients. CIMT and MoCA are valuable, non-invasive tools for early detection. Clinicians should adopt proactive screening strategies and address modifiable risk factors to mitigate long-term vascular and neurocognitive sequelae in lupus patients.
